# BK virus-associated collecting duct carcinoma of the renal allograft in a kidney-pancreas allograft recipient

**DOI:** 10.18632/oncotarget.24552

**Published:** 2018-02-22

**Authors:** Myriam Dao, Adrien Pécriaux, Thomas Bessede, Antoine Dürrbach, Charlotte Mussini, Catherine Guettier, Sophie Ferlicot

**Affiliations:** ^1^ Pathology Department, CHU Bicêtre, Le Kremlin-Bicêtre, France; ^2^ University Paris-Sud, Le Kremlin-Bicêtre, France; ^3^ Urology Department, CHU Bicêtre, Le Kremlin-Bicêtre, France; ^4^ Nephrology Department, CHU Bicêtre, Le Kremlin-Bicêtre, France

**Keywords:** collecting duct carcinoma, renal allograft carcinoma, kidney transplantation, BK polyomavirus, SV40

## Abstract

BK polyomavirus (BKV) nephropathy is a major concern in renal transplantation. Its main consequence is graft loss, which occurs in more than 50% of the cases. *De novo* renal cell carcinoma in renal allograft is a very rare event. Most of these tumors are papillary or clear cell carcinomas. We report herein the first case of collecting duct carcinoma of the renal allograft in a kidney-pancreas allograft adult recipient. Collecting duct carcinoma occurs long after the cure of a BKV nephropathy. At this time, BKV viremia and viruria were negative as well as the immunostaining for SV40 in the non-tumor kidney. The viral oncoprotein Tag persists only in the tumor cells. To preserve pancreas graft function, we maintained immunosuppression levels. After a 9-months follow-up, the evolution was free from clinical and radiological progression. The oncogenic role of BKV remains controversial in human cancers. However, strong experimental data have shown an association between BKV infection and urologic neoplasms. Further works might precise the exact role of polyomaviruses in renal carcinogenesis. In the meantime, clinical vigilance for early diagnostic of these tumors is mandatory after BKV nephropathy.

## INTRODUCTION

BK polyomavirus (BKV) infection represents a major concern in renal transplantation. The prevalence of BKV nephropathy in kidney allograft recipient is currently about 4% [1]. The main consequence of BKV nephropathy is graft loss which occurs in more than 50% of the cases. The oncogenic role of BKV has been controversial in human cancers. However, strong experimental data have shown an association between BKV infection and urologic neoplasms, especially high grade urothelial neoplasms [2].

Solid organ allograft recipients have an increased incidence of malignancies. The rate of renal cell carcinoma (RCC) among all post-transplantation malignancies is less than 5% after renal transplantation. Most of RCC are found in the recipient’s native kidneys and *de novo* RCC in renal allograft is a very rare event [3–7]. Most of these tumors are papillary or clear cell carcinomas.

Collecting duct carcinoma (CDC) of Bellini is a rare variant of renal cell carcinoma, with an aggressive behavior. Herein, we report the first case BKV-associated collecting duct carcinoma (CDC) of the renal allograft in an adult kidney-pancreas allograft recipient.

## CASE REPORT

A 39-year-old man received a combined kidney and pancreas transplantation from a deceased donor in 2005 for end-stage renal disease due to type 1 diabetes mellitus, diagnosed at the age of 9. Additional past medical history included high blood pressure, malaria, pleural and lymph node tuberculosis in 2004. At the time of the transplantation in 2005, he received methylprednisolone 500 mg once daily for 3 days post-transplant and rabbit-antithymocyte globulin 7 mg/kg daily for 4 days post-transplant. Initial maintenance immunosuppression included prednisone 5 mg once daily, mycophenolate mofetil (MMF) 500 mg twice daily and tacrolimus with target tacrolimus levels of 8–10 ng/mL. A renal allograft biopsy was performed 13 days after the transplantation because of delayed graft function. Histological analysis concluded to borderline changes with foci of tubulitis (t1) and minor interstitial infiltration (i1) according to the Banff classification. The patient was treated with methylprednisolone 500 mg bolus once daily for 3 days. The creatininemia-nadir was 106 µmol/L. Two years later, in 2007, the post-transplant course was complicated by biopsy-proven BKV nephropathy that subsequently resolved by a switch from MMF to Leflunomide 30 mg daily. BKV titers during follow-up remained undetectable or low-level (<2.30 log). Renal function remained stable thereafter, defined by creatininemia around 160 µmol/L, and pancreatic graft was functional without need for insulin. In 2016, systematic ultrasound examination disclosed an asymptomatic 3.5 cm-large tumor in renal allograft medulla. Previous renal allograft echography performed in 2015 was normal. F-18-fluoro-2-deoxyglucose (FDG) positron emission tomography in combination with computed tomography (PET-ct) highlighted a peripheral hypermetabolism of the renal tumor, without evidence of any extrarenal tumor. At this time, BKV viremia titers remained unchanged.

A tumor guided-biopsy was performed in November 2016. Histological analysis showed only rare malignant cells on a necrotic background not allowing a precise diagnosis. Graft biopsy of non-tumor kidney concurrently evidenced marked interstitial fibrosis with tubular atrophy (IF/TA), severe lesions of arteriolar hyaline thickening and focal segmental glomerulosclerosis, without any feature of polyomavirus associated nephropathy. Immunohistochemical study with SV40 antibody was negative in non-tumor kidney.

A radical kidney transplantectomy was performed in March 2017, in the extracapsular plane.

**Figure 1:**
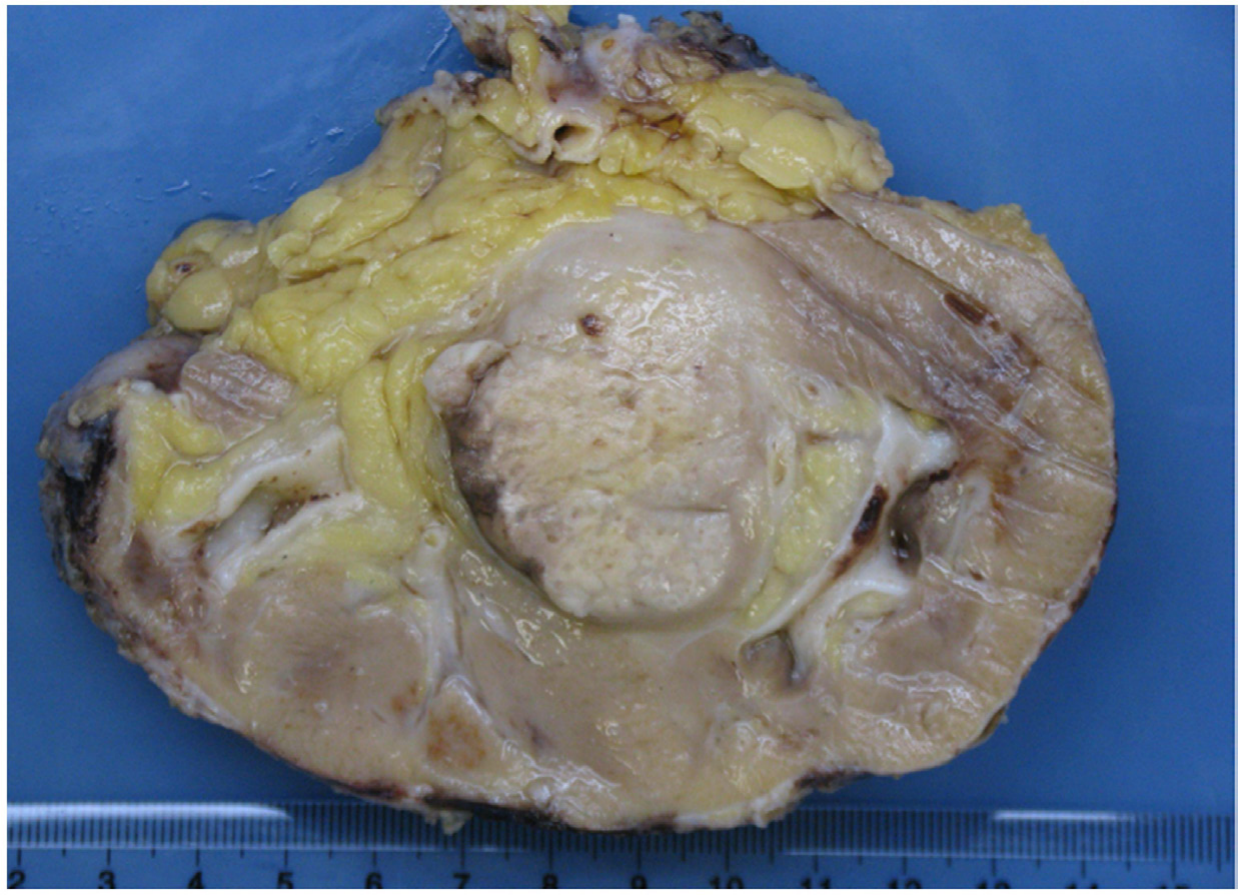
Gross examination of explanted kidney revealed an infiltrating white-gray solid tumor in the renal medulla

Gross examination (Figure 1) revealed an infiltrative white-gray solid tumor of the renal medulla, which measured 5.4 cm in its greatest dimension. Necrosis was evaluated around 50% of the entire tumor. Renal capsule was intact.

**Figure 2:**
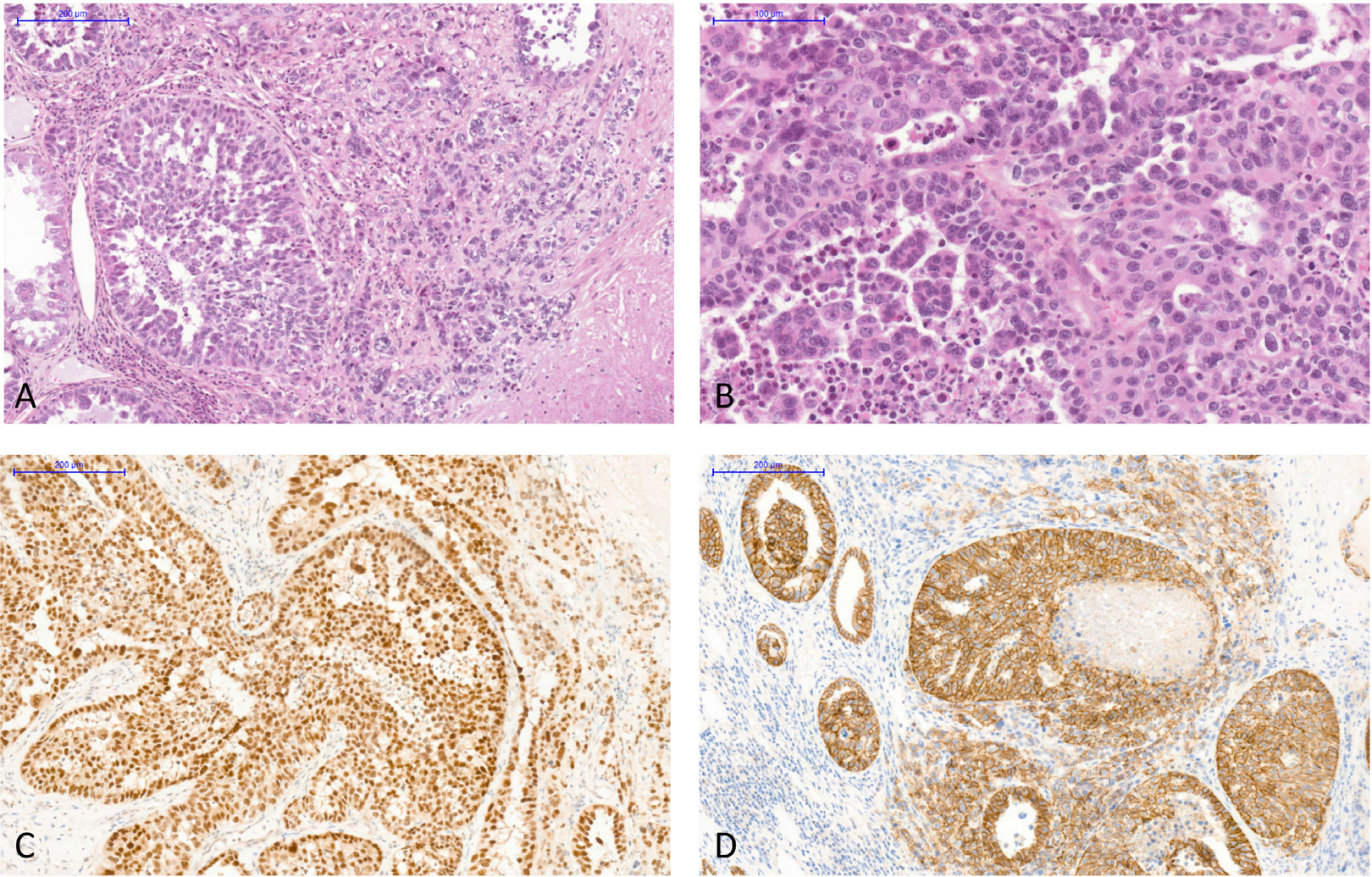
The allograft tumor exhibited histological features of collecting duct carcinoma of Bellini and immunohistochemistry staining excluded differential diagnoses (**A**) Various histological patterns were observed: solid, cribriform intermixed with independent cells in a desmoplastic stroma (hematoxylin, eosin and saffron, original magnification X100). (**B**) Tumor cells had abundant eosinophilic cytoplasm and large irregular nuclei with prominent nucleoli (hematoxylin, eosin and saffron, original magnification X200). (**C**) Immunohistochemistry with an antibody anti-PAX8, X100, showed strong intranuclear staining in tumor cells. (**D**) Immunohistochemistry with an antibody anti-E-cadherin, X100, showed strong membranous positivity of tumor cells.

Histologically, the tumor architecture was mainly solid and cribriform intermixed with trabecular, tubular, micropapillary and single cell patterns within a fibrous stroma (Figure 2). Tumor cells had abundant eosinophilic cytoplasm and large irregular nuclei with prominent nucleoli. Mitotic activity was low. The tumor was ill-circumscribed and infiltrated the adjacent renal parenchyma with an intraductal extension. Hilar lymph nodes were not metastatic. The urothelium was normal. The tumor was classified as collecting duct carcinoma and tumor stage was pT1b pNx according to the 2002 kidney cancer TNM staging system adapted for kidney graft tumor [4].

**Figure 3:**
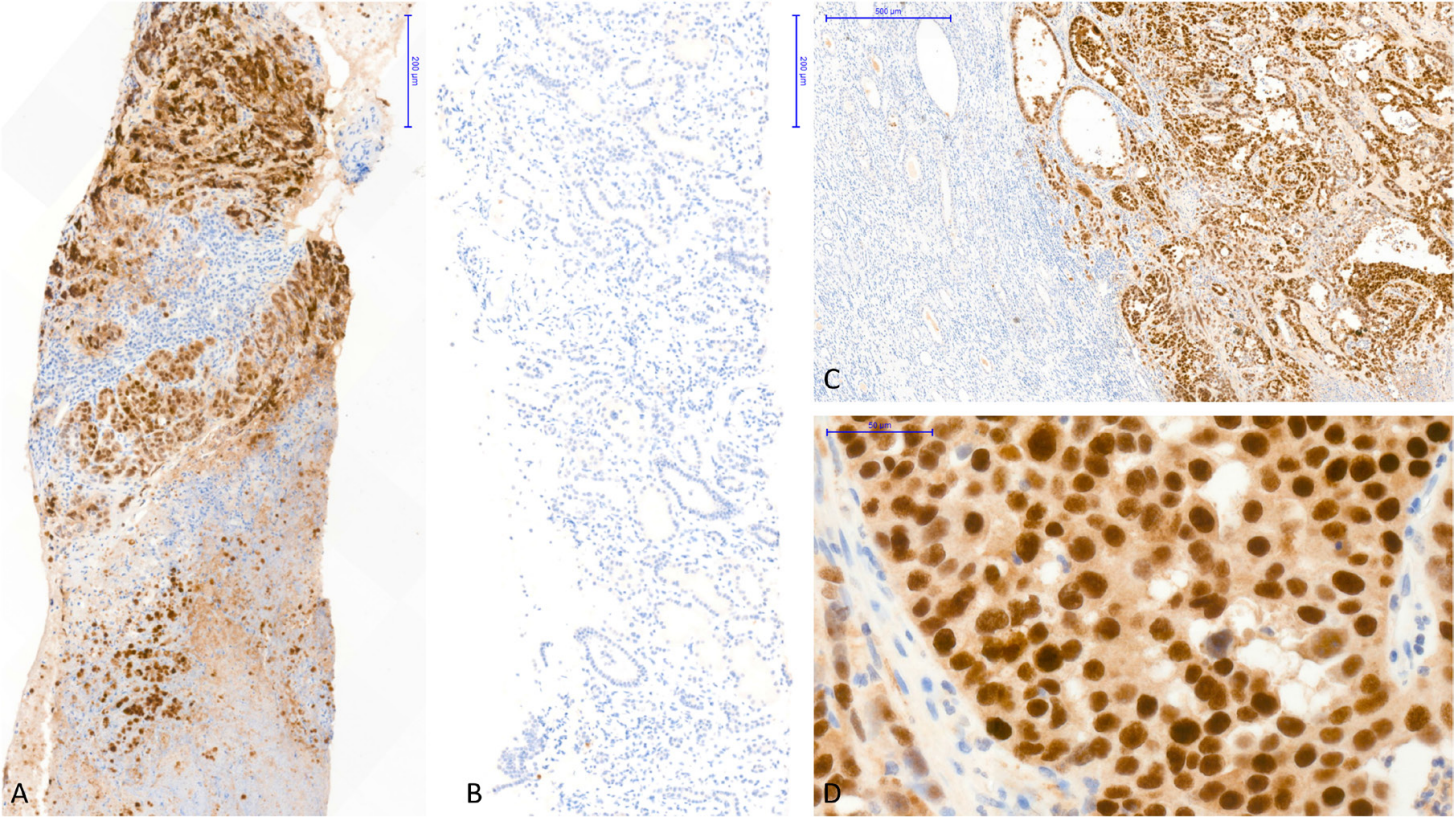
Immunohistochemistry staining showed strong SV40 expression in the tumor cells without polyomavirus replication in the non-tumor kidney (**A**) Immunohistochemistry with an antibody anti-SV40 (SV40 IHC), performed on the tumor guided-biopsy, showed strong nuclear staining in all tumor cells (X10). (**B**) SV40 IHC, performed concurrently on the non-tumor kidney biopsy, was completely negative (X10). (**C**) SV40 IHC, performed in radical kidney transplantectomy, showed strong nuclear staining in all tumor cells with a complete negativity of the non-tumor kidney (X5). (**D**) SV40 IHC showed strong nuclear staining in all tumor cells (X40). Abbreviation: SV40 IHC, immunohistochemistry with an antibody anti-SV40.

By immunohistochemistry, tumor cells were strongly and diffusely positive for PAX8, E-cadherin, CK7, INI1, and focally with CA9 and vimentin (Figure 2). No staining was observed for CK20, GATA3 and p504S. SV40 was expressed in the nuclei of all tumor cells (Figure 3).

Non-tumor allograft parenchyma showed no evidence of BKV nephropathy and was totally negative for SV40 immunostaining (Figure 3). Some glomeruli were ischemic without allograft glomerulopathy. IF/TA was discrete. Vascular lesions were marked with intimal fibrosis occlusion between 25 and 50% of the lumen (cv2) and arteriolar hyalinosis (ah3).

After the transplantectomy, an immuno-oncologic discussion has risen for this patient regarding his remaining pancreatic transplant at a multidisciplinary committee. On the one side, diagnosing a malignant disease in a solid organ recipient was exhorting to perform a pancreatic transplantectomy in order to discontinue his immunosuppressive treatment. On the other side, we have considered that: 1/ pancreatic transplantectomy is a highly morbid procedure, 2/ prognosis of CDC is spontaneously poor, even without immunosuppressive treatment, 3/ metastatic progression risk is not known when primary tumor has occurred in a transplant. No adjuvant anti-cancer treatment was decided, the pancreatic graft was left in place and the immunosuppressive treatment was continued. To date, with a 9-months follow-up, the evolution is free from clinical and radiological progression.

## DISCUSSION

Although the risk of RCC in native kidneys among renal allograft recipients is 15-100 times higher than in the general population [3], *de novo* RCC in renal allograft is a very rare event. In 1995, Penn *et al.* [7] from the CTTR (Cincinnati Transplant Tumor Registry) reported 24 cases among 7596 cancers in kidney allograft recipients from 1968 until 1994. The predominant histological subtypes of these kidney allograft cancers are papillary and clear cell RCC. Tillou *et al.* [4] analyzed RCC in kidney allograft recipients through a French renal transplant cohort. Seventy-nine renal allograft tumors were identified among 41 806 recipients. Forty-four were papillary RCC, 32 were clear cell RCC and 3 had mixed features fitting with the clear cell papillary RCC of the last WHO classification [8]. Barama *et al.* [5] analyzed 1073 patients who received a kidney graft in a single center from 1969 and 2002. Since 1991, 5 kidney allograft tumors were diagnosed. Four tumors were clear cell RCC and one was a papillary RCC. Patients from all these studies had an excellent survival after simple surgical treatment, whether after nephron sparing surgery or graft nephrectomy [3–6]. CDC of Bellini as observed in our patient is an uncommon variant of RCC, which represents less than 1% of all RCC. It is characterized by a highly aggressive behavior with a poor prognosis and a median survival of 30 months [9] even after surgical treatment.

Literature search revealed only few previously reported cases of CDC occurring in transplant recipients [10–14], which are presented in Table 1. Three of them occurred in pediatric recipients (two renal graft recipients [10,11] and one lung transplant recipient [12]). All were associated with active BKV nephropathy and immunohistochemistry highlighted strong nuclear expression of SV40 viral antigen by tumor cells. Three other cases of SV40-associated CDC were reported in adult kidney-graft recipients [11, 13, 14]. All patients with CDC occurring in renal graft were treated by transplantectomy and discontinuation of the immunosuppressive treatment. To preserve pancreas graft function, we decided to maintain immunosuppression levels. To date, with a 9-months follow-up, the evolution is free from clinical and radiological progression. Pancreatic graft remains functional without need for insulin.

**Table 1:** Immunosuppressive treatment and outcome of BKV-associated CDC in the literature

Author	Age at Tx (years)/Sex	Time BKVN (years) after Tx	Time Tumor (years) after Tx	Management of immunosuppressive treatment	Outcome of the patient
Emerson [10]	6.5/M	0.5	3.5	Discontinuation	No evidence of metastases or recurrence at the time of the report.
Dufek^a^ [12]	8/M	2	> 2	Switched to everolimus, reduction of tacrolimus and prednisone, discontinuation of MMF.	The patient died one week after the diagnosis was confirmed.
Gupta [11]	10/H 41/F	NR	NR	NR	NR
Kenan [13]	62/M	1	6	Discontinuation (?) after graft nephrectomy.	No evidence of metastases or recurrence at the time of the report.
Veldhuijzen [14]	62/F	0.5	4.5	Discontinuation.	The patient received second kidney graft 2.5 years after transplantectomy^b^.
Current study^c^	39/H	2	11	Continuation of immunosuppressive treatment by prednisone, leflunomide and tacrolimus.	No evidence of metastases or recurrence at the time of the report (6 months follow-up).

^a^Pediatric lung transplant recipient who developed BKV nephropathy of the native kidney, leading to end-stage renal failure and to CDC of the native kidney.

^b^2.5 years after transplantectomy, the patient received a second kidney transplant. Immunosuppressive regimen consists of prednisolone, mycophenolate and everolimus. After a follow-up of 24 months, the patient is doing well without BK viremia or tumor recurrence.

^c^Kidney-pancreas allograft recipient.

Abbreviations: BKVN, BK polyomavirus-associated nephropathy; CDC, collecting duct carcinoma; F, female; M, male; MMF, mycophenolate mofetil; NR, non-reported; Tx, transplantation.

The BKV belongs to the human polyomavirus family. A potential role of the polyomaviruses in variety of human cancers has been emphasized, including brain, pancreas, lung, liver, colon and urinary tract [15]. The mechanism of malignant transformation by the polyomaviruses is thought to be due to the presence of potent transforming genes. The polyomaviruses BKV, JCV and SV40 encode 2 viral oncogenes, the large T antigen (Tag) and the small t antigen (tag). Both viral oncoproteins can transform animal or human cells [16]. Other *in vivo* and *in vitro* studies have shown that the urothelium-specific expression of polyomavirus SV40 TAg in transgenic mice produced tumors strongly resembling human carcinoma *in situ* of the bladder [17, 18]. In human neoplasia, the role of BKV remains controversial [8]. However, several recent reports suggest an association between polyomavirus infection and urothelial or renal cell carcinoma in renal allograft recipients, affecting either the transplanted organ or the genito-urinary tract of the recipient [10, 12, 19, 20]. The oncogenic properties of BKV is linked to the ability of BKV Tag to inactivate regulators of cell cycle control including the pRB and p53 family proteins [21, 22]. Moreover, the BKV can induce chromosomal aberrations in human cells after integration into human chromosomal DNA [16].

In our patient, as in the recent reports from Kenan [13] and Veldhuijzen [14], the kidney allograft CDC occurred long after the transplantation and many years after the episode of BKV nephropathy. At the tumor time, BKV viremia and viruria were negative as well as the immunostaining for SV40 in the non-tumor kidney. The persistent expression of TAg in the tumor cells together with its negativity in the non-tumor kidney we observed in our patient strongly suggests that this protein exerts an oncogenic effect rather than being a by-stander [13]. Kenan *et al.* [13] proposed that the lack of polyomavirus viremia at time of tumor diagnostic and thereafter is due to latent infection with sporadic viral integration into the human genome which lead to neoplastic transformation. Conversely, viremia is thought to be due to “productive infection” resulting in cell death or cell lysis due to release of mature daughter virions from the infected host cells.

In conclusion, this case of BKV-associated CDC of the renal allograft is the first one described in a kidney-pancreas allograft adult recipient with a favorable outcome despite the continuation of the immunosuppressive treatment. A main point of interest is the occurrence of the tumor 9 years after the diagnosis and the cure of a BKV nephropathy with the persistence of the viral oncoprotein Tag only in the tumor cells.

From a practical point of view, clinical vigilance for early diagnosis of these allograft tumors is mandatory after BKV nephropathy.

## MATERIALS AND METHODS

Kidney allograft biopsies and kidney allograft explant were processed for routine light microscopy. Biopsy samples were fixed in AFA (Formalin, acetic acid and alcohol) for 6 hours. Kidney allograft was fixed in 10% formalin for 24 hours then sampled. Samples were paraffin-embedded and sliced 3 µm thick. Slides were stained with HES (hematoxylin, eosin and saffron), Masson trichrome, periodic acid Schiff and Jones methenamine silver.

Immunohistochemical staining was performed using the following commercially available antibodies by a Leica BOND-MAX™ autostainer (Leica Biosystems Newcastle Ltd, UK): mouse anti-BAF47/INI1 (BD Biosciences, USA; dilution 1/50), mouse anti-cytokeratin (CK) 7 (DakoCytomation, Denmark; dilution 1/800), mouse anti-CK20 (DakoCytomation, Denmark; dilution 1/100), polyclonal rabbit anti-PAX8 (Zytomed Systems Gmbh, Berlin, Germany; dilution 1/50), mouse anti-E-cadherin (InVitrogen, Carlsbad, USA; dilution 1/25), mouse anti-vimentin (DakoCytomation, Denmark; dilution 1/200), polyclonal rabbit anti-CA9 (Novus Biologicals, Littleton, USA; dilution 1/800), mouse anti-GATA3 (BioCare Medicals, USA; dilution 1/500), polyclonal rabbit anti-p504S (BioCare Medicals, USA; ready-to-use pre-diluted) and SV40 (Roche Ventana, USA; ready-to-use pre-diluted). Appropriate positive and negative controls were run concurrently for all the markers tested. Epitope retrieval was achieved using the ready-to-use Bond Epitope Retrieval Solution 1 (Leica Biosystems Newcastle Ltd, UK).

## Abbreviations

BKV: BK polyomavirus
CDC: collecting duct carcinoma
CK: cytokeratin
FDG: F-18-fluoro-2-deoxyglucose
IF/TA: interstitial fibrosis and tubular atrophy
MMF: mycophenolic mofetil
PET-ct: positron emission tomography in combination with computed tomography
RCC: renal cell carcinoma
SV40 IHC: immunohistochemistry with an antibody anti-SV40
tag: small t antigen
Tag: large T antigen
